# IBGJO: Improved Binary Golden Jackal Optimization with Chaotic Tent Map and Cosine Similarity for Feature Selection

**DOI:** 10.3390/e25081128

**Published:** 2023-07-27

**Authors:** Kunpeng Zhang, Yanheng Liu, Fang Mei, Geng Sun, Jingyi Jin

**Affiliations:** 1 College of Computer Science and Technology, Jilin University, Changchun 130012, China; zhangkp18@mails.jlu.edu.cn (K.Z.); yhliu@jlu.edu.cn (Y.L.); jyjin20@mails.jlu.edu.cn (J.J.); 2 Key Laboratory of Symbol Computation and Knowledge Engineering of the Ministry of Education, Jilin University, Changchun 130012, China

**Keywords:** feature selection, machine learning, classification, chaotic, cosine similarity, golden jackal optimization

## Abstract

Feature selection is a crucial process in machine learning and data mining that identifies the most pertinent and valuable features in a dataset. It enhances the efficacy and precision of predictive models by efficiently reducing the number of features. This reduction improves classification accuracy, lessens the computational burden, and enhances overall performance. This study proposes the improved binary golden jackal optimization (IBGJO) algorithm, an extension of the conventional golden jackal optimization (GJO) algorithm. IBGJO serves as a search strategy for wrapper-based feature selection. It comprises three key factors: a population initialization process with a chaotic tent map (CTM) mechanism that enhances exploitation abilities and guarantees population diversity, an adaptive position update mechanism using cosine similarity to prevent premature convergence, and a binary mechanism well-suited for binary feature selection problems. We evaluated IBGJO on 28 classical datasets from the UC Irvine Machine Learning Repository. The results show that the CTM mechanism and the position update strategy based on cosine similarity proposed in IBGJO can significantly improve the Rate of convergence of the conventional GJO algorithm, and the accuracy is also significantly better than other algorithms. Additionally, we evaluate the effectiveness and performance of the enhanced factors. Our empirical results show that the proposed CTM mechanism and the position update strategy based on cosine similarity can help the conventional GJO algorithm converge faster.

## 1. Introduction

Machine learning and data mining have expanded in many fields, including active matter, molecular and materials science, nature language process (NLP) and biomedicine [1,2,3]. To create more complex machine learning models, many datasets with high-dimensional feature spaces are created [4,5]. However, as the dimensionality of data increases, there are more and more redundant features, and it becomes more difficult to train models with high generalization ability. Therefore, it is necessary to perform feature selection to solve these problems. Feature selection is a critical step in data mining and machine learning that involves identifying the most relevant and useful features within a dataset or set of characteristics. Predictive models can be more effective and precise by eliminating redundant or unnecessary features. This improves classification accuracy and helps algorithms generalize better to new data, prevent overfitting, and produce more accurate predictions. In addition to these benefits, feature selection can help uncover hidden relationships within the data and provide more insightful explanations for predictive models.

Unsupervised Feature Selection methods can be classified into three main approaches, similar to supervised and semi-supervised feature selection [6,7]. These approaches are determined by the feature selection strategy employed, including filter, wrapper, and embedded methods [8,9]. In particular, filter methods rank the features according to the calculated scores by using a statistical metric to assign each feature a meaningful score. However, they might use up more computational resources. In the wrapper method, the selection subset obtained by the selection algorithm is evaluated using a classifier, and feature selection is guided by the feedback of the classifier [10]. As a result, the accuracy of the wrapper method is greater than the filtering method, as indicated by [11]. Furthermore, feature selection is regarded as a component of the machine learning training phase, which makes embedding methods a particular case of packing methods [12,13].

Meanwhile, it is possible to think of feature selection as a global group optimization problem. In particular, some of the original dataset answers the optimization problem, which can be resolved using exhaustive and heuristic search techniques [14]. In contrast to heuristic search methods, exhaustive search methods typically have higher computational costs, especially for high-dimensional datasets [4]. Using meta-heuristic search techniques may be a more practical way to solve the feature selection problem [14]. Therefore, it is essential to choose features using efficient methods.

Evolutionary algorithms (EAs) have recently been used to solve feature selection challenges for the global search capacity of feature selection methods. Numerous researchers have used various population intelligence techniques to address feature selection issues, including the cuckoo search (CS) [15], genetic algorithm (GA) [11], particle swarm optimization (PSO) [12], whale optimization algorithm (WOA) [16], sparrow search algorithm (SSA) [17], harris hawks optimization (HHO) [18,19] and variants of these algorithms, for dealing with the feature selection problems. For instance, Hegazy et al. [20] attempt to enhance the basic SSA structure to increase the solution accuracy, reliability, and convergence speed. Additionally, Behrouz et al. propose an unsupervised probabilistic feature selection algorithm using ant colony optimization [21].

The golden jackal optimization (GJO) algorithm is one of the EAs, and research has demonstrated that it is both efficient and simple to apply [22]. Nevertheless, despite its extensive use, the conventional GJO algorithm may have certain drawbacks, such as insufficiently exploiting issue areas. In addition, the no free lunch (NFL) theory contends that no single algorithm is capable of solving every optimization problem [23]. The conventional GJO algorithm was created to solve continuous optimization issues. There might be better choices for feature selection tasks involving binary solution spaces. These circumstances drive us to improve the conventional GJO to make it better suited for feature selection tasks.

The main contributions of this paper are summarized as follows:

- We aim to simultaneously reduce the number of selected features and improve the classification accuracy. Specifically, we design a fitness function to achieve these optimization objectives jointly.
- We propose an improved binary golden jackal optimization algorithm (IBGJO) to solve the designed fitness function. First, IBGJO introduces a chaotic tent map to improve the exploitation capability of conventional GJO. Second, a new position-updating mechanism by cosine similarity is proposed to balance the exploitation and exploration capabilities of the algorithm. Finally, a binarization strategy is introduced to transfer the continuous solution space to the binary ones, making it suitable for dealing with feature selection solutions.
- We conduct various experiments to assess the performance of the proposed IBGJO with the comparative algorithms on 28 classical UC Irvine (UCI) Machine Learning Repository datasets in terms of average fitness value, average classification accuracy, average CPU running time and average number of selected features.

The rest of this paper is organized as follows. Section 2 gives a brief overview of the related work. Section 3 designs the formulated fitness function of feature selection. Section 4 gives the details of IBGJO. Section 5 presents the experimental results. Finally, Section 6 concludes this paper and suggests the future works.

## 2. Related Work

The significance of wrapper-based selection techniques in feature selection optimizations cannot be overlooked [24,25]. These methods operate on the premise of treating feature selection as a black box, and employ meta-heuristic algorithms and classifiers to obtain the optimal subset [26]. Numerous classical meta-heuristic algorithms have undergone modifications to tackle the feature selection problem, such as binary bat algorithm (BBA) [27], bare bones particle swarm optimization algorithm (BPSO) [12], binary gray wolf optimization algorithm (BGWO) [28], binary gravitational search algorithm (BGSA) [29], and so on.

In recent times, an increasing number of novel algorithms have been proposed to enhance the optimization of feature selection problems based on the wrapper approach, due to their vital significance. For instance, Al-Tashi et al. [30] examine binary optimization utilizing hybrid grey wolf optimization for feature selection in their paper. To resolve feature selection issues, a binary version of the hybrid grey wolf optimization (GWO) and PSO is suggested. In 2019 [31], binary variations of the butterfly optimization algorithm (BOA) are suggested and utilized to choose the best feature subset for classification purposes. A self-adaptive particle swarm optimization (SaPSO) approach is suggested by Xue et al., especially for large-scale feature selection [32]. The two-archive multi-objective artificial bee colony algorithm (TMABC-FS) is a multi-objective feature selection approach that Zhang et al. investigate to satisfy diverse decision-makers’ criteria [33]. To increase the predictability of the hospitalization expense model, a novel method proposed based on the GA for feature selection and parameter optimization of the support vector machine (SVM) in 2019 [34]. Aimed at finding distinguishing characteristics across several class labels, Zhang et al. [35] offer an embedded multi-label feature selection approach with manifold regularization. To develop a more affordable computational model for voice analysis-based emotion categorization, Dey et al [36] offer a meta-heuristic feature selection (FS) method employing a hybrid of equilibrium optimization (EO) and golden ratio optimization (GRO) algorithms. Wang and Chen [37] propose an improved whale optimization algorithm (CMWOA) that integrates chaotic and multi-swarm techniques to accomplish parameter optimization and feature selection simultaneously for SVM in 2020. For feature selection issues in medical diagnosis, a hybrid crow search optimization method integrated with chaos theory and fuzzy c-means algorithm was proposed in 2020 [38].

Several leading-edge researchers have focused on GJO algorithms for optimizing feature selection. Initially designed to address continuous problems, GJO requires transfer functions to convert it into a binary form (BGJO) [39] that can effectively handle feature selection optimizations. While some studies have made strides in addressing feature selection challenges across a variety of contexts, it is important to note that the NFL [23] theorem holds that no method can solve every optimization problem. Furthermore, none of the aforementioned research has identified optimal subsets of variables across all datasets tested. Nonetheless, given the strong potential of conventional GJO in this area, our aim in this study is to incorporate several enhanced factors into conventional GJO with the objective of improving the efficiency of feature selection optimizations.

## 3. Problem Formulation

In this study, feature selection aims to minimize the number of chosen features while improving the classification accuracy, which can be defined as a multi-objective optimization problem [40]. To consider the two objectives of optimization, we constructed the following fitness function:

(1) $$f_{Fitness}=\alpha \cdot E_{r}+\beta \cdot \frac{F_{s}}{F_{a}}$$

where $F_{s}$ and $F_{a}$ stand for the number of chosen features and the total number of features, respectively, and $E_{r}$ is the classification error rate of a certain classifier. Additionally, the weights used to balance these two goals are α and β.

The formulated feature selection problem has a nonlinear discrete search space with numerous potential local minimum points. As a result, we suggest the binary IBGJO algorithm to address the feature selection problem.

## 4. Proposed Improved Golden Jackal Optimization Algorithm for Feature Selection

The following section provides a succinct overview of the conventional GJO algorithm and its key principles. Additionally, the conventional GJO algorithm is reviewed before delving into a comprehensive discussion of the proposed IBGJO algorithm’s formulation.

### 4.1. Conventional Golden Jackal Optimization

**Figure 1:**
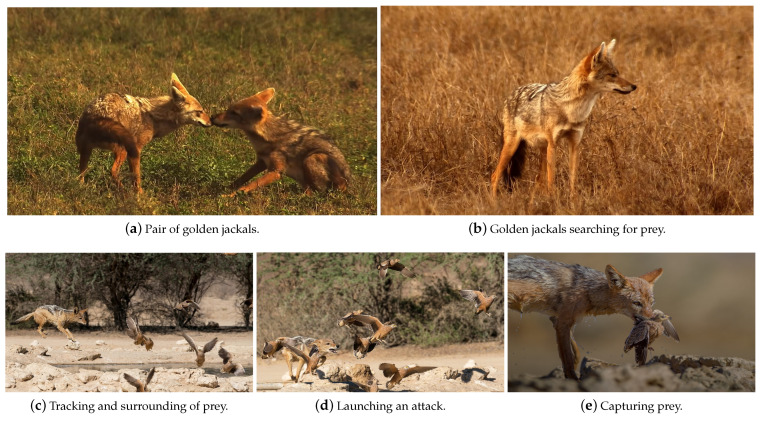
The stages of golden jackal pair hunting.

The conventional GJO algorithm is inspired by the hunting behavior of golden jackal pairs and adopts a swarm-based approach [22]. Figure 1 shows the entire foraging process of the golden jackal pair. The whole foraging process includes searching for prey, tracking and surrounding of prey, attacking prey and capturing prey. This section delves into the mathematical modeling of the conventional GJO algorithm.

#### 4.1.1. Search Space Formulation

The initial solution of the golden jackal optimization algorithm is also uniformly distributed on the search space, which is similar to other metaheuristic methods, and its distribution is as follows:

(2) $$Y_{0}=Y_{\min}+rand\times \left(ub-lb\right)$$

where $Y_{0}$ represents the initial randomized population, and ub and lb denote the upper and lower boundaries of the decision variables. Moreover, rand is a random number that falls within the range of [0,1]. The initialization procedure involves generating a foundational Prey matrix, with the male and female jackals occupying the first and second positions, correspondingly. The composition of the Prey is illustrated as follows:

(3) $$Prey= [\begin{array}{cccc} Y_{1,1} & Y_{1,2} & \cdots & Y_{1,d}\\ Y_{2,1} & Y_{2,2} & \cdots & Y_{2,d}\\ : & : & : & :\\ Y_{n,1} & Y_{n,2} & \cdots & Y_{n,d} \end{array}]$$

where $Y_{ij}$ stands for the *i*-th prey’s *j*-th dimension. There are *n* preys and *d* variables in total. The prey position can be regarded as an optimal solution. During optimization, an objective function is used to assess the fitness of each prey, with the resulting fitness values being compiled into a matrix:

(4) $$F_{OA}= [\begin{array}{c} f(Y_{1,1};Y_{1,2};\cdots ;Y_{1,d})\\ f(Y_{2,1};Y_{2,2};\cdots ;Y_{2,d})\\ :\\ f(Y_{n,1};Y_{n,2};\cdots ;Y_{n,d}) \end{array}]$$

where *f* is the objective function, $Y_{ij}$ displays the value of the *j*-th dimension of the *i*-th prey, and $F_{OA}$ is the matrix for storing each prey’s fitness. A male jackal (MJ) is the most suitable, and a female jackal (FMJ) is the second most suitable. The jackal couple finds the appropriate prey location.

#### 4.1.2. Searching for Prey (Exploration Stage)

With their remarkable capability to detect and pursue prey, jackals can usually track down food successfully. Nevertheless, there are instances when their attempts fail, and the potential prey evades capture, prompting the jackals to give up and search for alternative sources of sustenance. During hunts, the MJ takes the lead, while the FMJ follows closely behind, and the mathematically modelled jackal pairs hunt as follows:

(5a) $$Y_{1}\left(t\right)=Y_{M}\left(t\right)-E.\left|Y_{M}\left(t\right)-rl.Prey\left(t\right)\right|$$

(5b) $$Y_{2}\left(t\right)=Y_{FM}\left(t\right)-E.\left|Y_{FM}\left(t\right)-rl.Prey\left(t\right)\right|$$

where *t* represents the current iteration, Prey(t) is the vector indicating the prey’s position. In contrast, YM(t) and YFM(t) are the positions of the MJ and FMJ, respectively. The revised positions of MJ and FMJ in relation to the prey are represented by $Y_{1}\left(t\right)$ and $Y_{2}\left(t\right)$. The prey’s evasive energy *E* is computed as:

(6a) $$E=E_{1}*E_{0}$$

(6b) $$E_{0}=2*r-1$$

(6c) $$E_{1}=c_{1}*\left(1-\left(t/T\right)\right)$$

$E_{0}$ depicts the beginning state of the prey’s energy, while $E_{1}$ represents the prey’s declining energy, where *r* is any random value between 0 and 1.

*T* stands for the max iteration number and $c_{1}$ is a constant value of 1.5. $E_{1}$ decreases linearly across iterations, from 1.5 to 0. In Equations (5a) and (5b), the distance between the jackal and the prey is calculated by |Y(t)−rl.Prey(t)|. Depending on how well the prey manages to evade the jackal, this distance is either added to or deducted from its present location. The vector of random numbers rl in Equations (5a) and (5b) represents the Lévy movement and is based on the Lévy flight. Prey is multiplied by rl to imitate Lévy-style prey movement, which is comparable to MPA [41] and is computed as follows:

(7) rl=0.05∗LF(y)

LF is the Lévy flight function, which is calculated as follows:

(8) $$LF\left(y\right)=0.01\times \left(\mu \times \sigma \right)/\left(\left|v^{\left(1/\beta \right)}\right|\right);\sigma ={\left(\frac{\Gamma (1+\beta )\times \sin(\pi \beta /2)}{\Gamma \left(\frac{1+\beta }{2}\right)\times \beta \times \left(2^{\frac{\beta -1}{2}}\right)}\right)}^{1/\beta }$$

where β is constant set to 1.5 and *u*, *v* are random values inside of (0,1). The jackal positions are updated by averaging Equations (5a) and (5b), which results in the following:

(9) $$Y\left(t+1\right)=\frac{Y_{1}\left(t\right)+Y_{2}\left(t\right)}{2}$$

#### 4.1.3. Tracking and Pouncing the Prey (Exploitation Stage)

As prey are pursued by jackals, their evasive energy declines, leading to the eventual encirclement of the prey by a pair of jackals identified in an earlier phase. Once encircled, the prey is attacked and consumed by the jackals. The following mathematical model is a representation of the hunting behaviour of male and female jackals that hunt in pairs, which is as follows:

(10a) $$Y_{1}\left(t\right)=Y_{M}\left(t\right)-E.\left|rl.Y_{M}\left(t\right)-Prey\left(t\right)\right|$$

(10b) $$Y_{2}\left(t\right)=Y_{FM}\left(t\right)-E.\left|rl.Y_{FM}\left(t\right)-Prey\left(t\right)\right|$$

where Prey(t) is the position vector of the prey during the current iteration *t*, and $Y_{M}\left(t\right)$ and $Y_{F}M\left(t\right)$ indicate the position of the MJ and FMJ. The updated MJ and FMJ positions in relation to the prey are represented by $Y_{1}\left(t\right)$ and $Y_{2}\left(t\right)$. Equation (6a) determines the prey’s evading energy, or *E*. The jackal positions are updated in accordance with Equation (9).

The purpose of rl in Equations (10a) and (10b) is to allow for arbitrary behavior in the exploitation stage, favoring exploration and avoiding local optima. Equation (7) is used to determine rl. In the final iterations, this component aids in avoiding local optima sluggishness.

As a result of jackals moving closer to the prey, the factor can be carefully considered. Typically, natural obstacles stand in the way of jackals’ proper and swift movement toward their prey. This is the goal of rl during the exploitation stage.

#### 4.1.4. Switching from Exploration to Exploitation

The escape energy of the prey is utilized in the conventional GJO algorithm to transition from exploration to exploitation. Throughout avoiding behavior, prey energy significantly decreases. In light of this, Equation (6a) is used to represent the evasive energy. Every repetition, the initial energy $E_{0}$ deviates arbitrarily from the range of −1 to 1. The prey is physically waning when $E_{0}$ value decreases from 0 to −1, but when $E_{0}$ value increases from 0 to 1, it indicates an improvement in the strength of prey.

**Figure 2:**
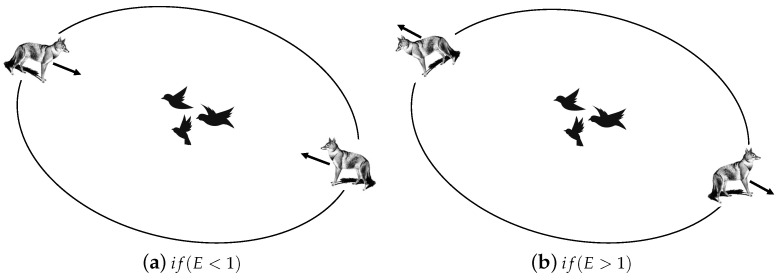
Attacking and searching for prey.

According to Figure 2, the altering avoiding energy *E* decreases over the iteration procedure. When |E|>1, jackal partners hunt for prey that is exploring in different areas, and when |E|<1, the jackal attacks the prey and engages in predation, as depicted in Figure 1.

To sum up, the conventional GJO search procedure starts with the random generation of a population of prey (possible solutions). MJ and FMJ hunting couples calculate the location of the prey during iterations. Each prospective member of the population updates their separation from the jackal pair. To emphasize exploration and exploitation, the $E_{1}$ parameter is decreased from 1.5 to 0, accordingly. When E>1, the hunting pair of golden jackals strays from their victim, and when E<1, it gathers at the prey. The conventional GJO algorithm is finally completed by satisfying an end criterion. Algorithm 1 presents the conventional GJO algorithm’s pseudo-code.
**Algorithm 1** Conventional Golden Jackal Optimization**Require:** The size of population $N_{pop}$, solution dimension $N_{dim}$, the max number of iterations $T_{max}$, lower and upper bounds $L_{b}$, $U_{b}$, the fitness function, the golden jackal GJ, prey, etc.**Ensure:** The best solution found in the search process 1: Initializing the population through random mechanism 2: **While**$\left(t<T_{max}\right)$ 3:   Calculate the fitness values of preys 4:   $Y_{1}=$ best prey (Male Jackal position) 5:   $Y_{2}=$ second best prey (Female Jackal Position) 6:   **for** (each prey) 7:      Update the evading energy *E* using Equations (6a), (6b) and (6c) 8:      Update rl using Equations (7) and (8) 9:      **if** (|E|>=1) // (Exploration phase)10:         Update the prey position using Equations (5b), (5a) and (9)11:      **if** (|E|<1) //(Exploitation phase)12:         Update the prey position using Equations (10a), (10b) and (9)13:   **end for**14:   t=t+115: **end While**16: **return $Y_{1}$**

### 4.2. The Proposed IBGJO

This section introduces the enhancement factors in the proposed IBGJO algorithm, including the random population initialization strategy based on the Chaotic Tent map, the optimal location update mechanism based on cosine similarity, and the sigmoid function used to discretization the continuous solution space problem. Finally, the complexity of the IBGJO algorithm was analyzed.

#### 4.2.1. Chaotic Tent Map for Initiate Population

**Figure 3:**
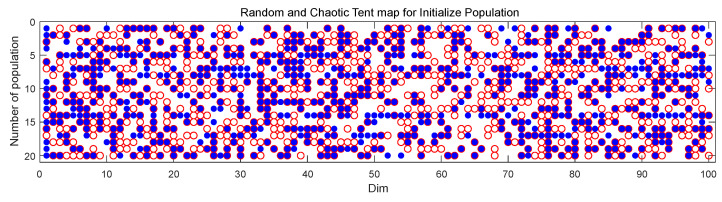
Random and CTM mechanisms for initialize population, where red dot represents the random mechanism, and blue represents CTM mechanism.

In the conventional GJO algorithm, initial population information is generated randomly, which can pose difficulties in retaining population diversity and hinder the algorithm’s effectiveness in achieving the optimal solution. In contrast, the chaotic tent map (CTM) mechanism is characterized by randomness, ergodicity, and regularity. It can be used either to generate the initial population or as a perturbation during the optimization process [37,42]. This approach overcomes the limitation of the algorithm becoming trapped in a suboptimal local solution, thereby improving its search efficiency compared to the original algorithm. The CTM mechanism is described as Algorithm 2.
**Algorithm 2** Chaotic Tent Map (CTM) MechanismDefine and initialize the related parameters: the size of population $N_{pop}$, solution dimension $N_{dim}$, chaotic tent map threshold *a*, low boundaries lb and up boundaries ub, respectively.
 1: **For** i=1 to $N_{pop}$ 2:   **For** j=1 to $N_{dim}$ 3:      **If** rand<a 4:         $x_{i,j}$ = $\frac{rand}{a}$ 5:      **Else** 6:         $x_{i,j}$ = a·(1−rand) 7:   $x_{i}$ = lb + $x_{i}\cdot \left(ub-lb\right)$ 8: return mean of *x* 9: **For** i=1 to $N_{pop}$10:   **For** j=1 to $N_{dim}$11:      **If** $x_{i,j}<mean$12:         $x_{i,j}$ = 013:      **Else**14:         $x_{i,j}$ = 115: return *x*
where *a* is the tent map’s call threshold, generally set as 0.5. In IBGJO, we use a CTM as the initialization mechanism. Considering the different dimensions of datasets, we provide Hillvalley in 28 datasets as an example of population initialization. As shown in Figure 3, the number of golden jackals in the population is 20, and the dimension is 100. And in Figure 3, the points labelled as random population initialization are denoted in red, while the points labelled as CTM population initialization are represented in blue. As can be seen, compared with the random mechanism, the CTM mechanism has good distribution and randomness. Therefore, the initialized population is more evenly distributed in the search space, which is more conducive to the algorithm’s optimization efficiency and solution accuracy.

#### 4.2.2. Cosine Similarity for Position Update

The conventional GJO algorithm (Algorithm 3) updates the position of jackals by Equation (9) during the iteration process, equivalent to using the mean as a more optimal solution. Although this method can ensure the smoothness of jackal position updates, it has some drawbacks. The most obvious flaw is that it does not consider the correlation between different features. When there is a correlation between features, using the mean update mechanism may lead to some features being overemphasized or ignored, thereby affecting the model’s performance. In addition, when the data distribution is uneven, using the mean update mechanism may lead to poor prediction performance of the model for specific data. Therefore, we propose cosine similarity for positions updating of FMJ and MJ. Compared with the mean update mechanism, the advantage of using cosine similarity as the update mechanism is that it can consider the correlation between different features, thus updating model parameters more accurately. In addition, cosine similarity is not affected by vector length and data distribution and is suitable for high-dimensional data [43]. The mathematical model of cosine similarity is defined as follows:

(11) $$Cos_{sim}\left(Y_{1}\left(t\right),Y_{2}\left(t\right)\right)=\frac{Y_{1}\left(t\right)\cdot Y_{2}\left(t\right)}{∥Y_{1}\left(t\right)∥∥Y_{2}\left(t\right)∥}$$

where the $Y_{1}\left(t\right)$ and $Y_{2}\left(t\right)$ represent the position of FMJ and MJ, respectively, and the · means dot product. $\left|\right|Y_{1}\left(t\right)\left|\right|$ and $\left|\right|Y_{2}\left(t\right)\left|\right|$ represent the lengths of FMJ and MJ, respectively. The value range of $Cos_{sim}\left(Y_{1}\left(t\right),Y_{2}\left(t\right)\right)$ is [−1, 1]. In this paper, we improve the cosine similarity between golden jackal pairs, using the absolute value as the weight of position update, which is defined as follows:

(12) $$Y\left(t+1\right)=Y_{1}\left(t\right)\times |Cos_{sim}\left(Y_{1}\left(t\right),Y_{2}\left(t\right)\right)|+Y_{2}\left(t\right)\times (1-|Cos_{sim}\left(Y_{1}\left(t\right),Y_{2}\left(t\right)\right)\left|\right)$$

**Algorithm 3** Improved Binary Golden Jackal Optimization**Require:** The size of population $N_{pop}$, solution dimension $N_{dim}$, the max number of iterations $T_{max}$, lower and upper bounds $L_{b}$, $U_{b}$, the fitness function, the golden jackal GJ, prey, etc.**Ensure:** The best solution found in the search process 1: Initializing the population through chaotic tent mechanism by Algorithm 2 2: **While**$\left(t<T_{max}\right)$ 3:   Calculate the fitness values of preys 4:   $Y_{1}=$ best prey (Male Jackal position) 5:   $Y_{2}=$ second best prey (Female Jackal Position) 6:   **for** (each prey) 7:      Update the evading energy *E* using Equations (6a), (6b) and (6c) 8:      Update rl using Equations (7) and (8) 9:      **if** (|E|>=1) // (Exploration phase)10:         Update the prey position using Equations (5b), (5a) and (12)11:      **if** (|E|<1) //(Exploitation phase)12:         Update the prey position using Equations (10a), (10b) and (12)13:   **end for**14:   t=t+115: **end While**16: **return $Y_{1}$**

#### 4.2.3. Binary Mechanism Sigmoid

**Figure 4:**
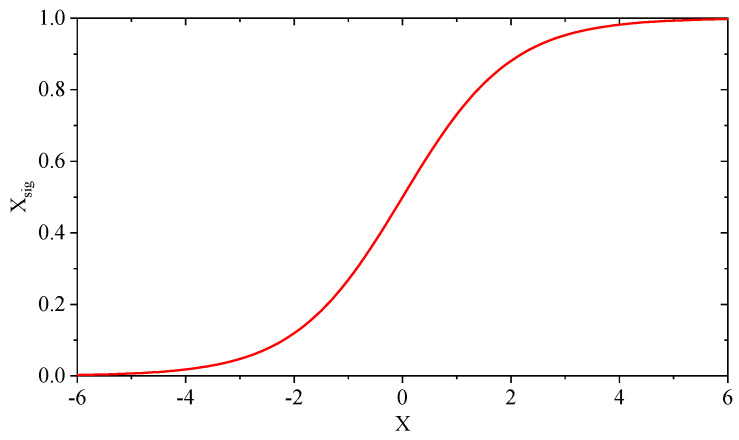
Sigmoid Binary transfer function.

The solutions in conventional GJO are continuous and can be updated using the Equations (5a), (5b), (10a) and (10b) directly. However, the solution space of the formulated feature selection problem is discrete, which cannot be handled by conventional GJO. Therefore, it is suitable for feature selection problems by introducing a binary mechanism to map the solutions from continuous to discrete space. For the solution mappings in this work, the commonly used *S*-shaped transfer function [44], i.e., the Sigmoid function, is applied to conventional GJO and IBGJO. The details of this function are as follows. Moreover, the binary mechanism is elucidated in Equations (13) and (14) as follows:

(13) $$x_{sig}=\frac{1}{1+e^{-x}},$$

(14) $$x_{binary}=\left\{\begin{array}{cc} 1, & N_{random}\leq x_{sig}\\ 0, & N_{random}>x_{sig} \end{array}\right.$$

where $x_{binary}$ is the converted binary solution of the feature selection problem, and $N_{random}$ is a random number used as the threshold. Figure 4 presents the binary mechanism that we used in this paper.

### 4.3. Feature Selection Based on IBGJO

A solution could be viewed as a golden jackal for the formulated feature selection problem when employing the suggested IBGJO. Consequently, the answer could be stated as follows:

(15) $$g=(G_{1},G_{2},G_{3}\ldots ,G_{N_{dim}})$$

where $N_{dim}$ represents the number of features while $N_{pop}$ is the number of individuals, thus, the IBGJO population is expressed as follows:

(16) $$\begin{array}{ccc} & pop & = [\begin{array}{c} g_{1}\\ g_{2}\\ \vdots \\ g_{N_{pop}} \end{array}]\\ & = & [\begin{array}{c} \begin{array}{ccccc} G_{1}^{1} & G_{2}^{1} & G_{3}^{1} & \cdots & G_{N_{dim}}^{1}\\ G_{1}^{2} & G_{2}^{2} & G_{3}^{2} & \cdots & G_{N_{dim}}^{2}\\ \vdots & \vdots & \vdots & \vdots & \vdots \\ G_{1}^{N_{pop}} & G_{2}^{N_{pop}} & G_{3}^{N_{pop}} & \cdots & G_{N_{dim}}^{N_{pop}} \end{array} \end{array}] \end{array}$$

### 4.4. Computational Complexity

The complexity of the conventional GJO algorithm depends on various factors, including the size of the individuals $N_{pop}$, and the number of iterations $T_{max}$. The exploration phase or exploitation phase is performed in each iteration. Therefore, the overall time complexity of conventional GJO consists of the exploration and exploitation phase. Thus, the overall time complexity of conventional GJO is given as follows:

(17) $$\begin{array}{cc} O(GJO) & =O(T_{max}\left(O\left(exploration\right)+O\left(exploration\right)\right))\\ \; & =O(T_{max}\left(N_{pop}\cdot N_{dim}+N_{pop}\cdot N_{dim}\right))\\ \; & =O(T_{max}\cdot N_{pop}\cdot N_{dim}) \end{array}$$

Since the structure of the proposed IBGJO is similar to conventional GJO, therefore, the computational complexity of IBGJO is also determined to be $O(T_{max}\cdot N_{pop}\cdot N_{dim})$. As a result, for a given feature selection problem, IBGJO does not require noticeably more computation time than conventional GJO, as both conventional GJO and IBGJO algorithms possess equivalent computational complexity. Notably, the average execution time of IBGJO in the experimental results is better than that of conventional GJO; this may be attributed to the enhanced factors employed in IBGJO, which will help improve the searchability of IBGJO and promote its fast convergence.

## 5. Experiments and Analysis

In this section, we conduct tests to evaluate the performance of the proposed IBGJO algorithm for dealing with feature selection problems. First, the datasets and setups used in the experiments are introduced. Then, the test results obtained by IBGJO and several comparison algorithms are presented and analyzed. Moreover, several other algorithms are selected for comparison.

### 5.1. Datasets and Setup

In this work, we provide the datasets used in this article and the parameter settings for the experiment.

#### 5.1.1. Benchmark Datasets

This section introduces the benchmark datasets used in different algorithms’ evaluations and parameter setups. Due to the fact that the UCI dataset covers multiple fields, such as Life, Social, Physical and so on, many research works use the UCI dataset as the benchmark data. For example, 10, 14, 16 and 20 datasets in the UCI dataset were respectively selected as experimental data in [21,45,46,47]. Therefore, the datasets used in our experiments refer to some datasets in their work and has been expanded to 28 datasets. By using these well-known datasets, we intended to facilitate comparisons with existing algorithms and provide a basis for future research. The primary information of these datasets is shown in Table 1.

#### 5.1.2. Experiment Setup

We compare IBMRFO with several other algorithms for feature selection experiments, including BCS, BGWO, BHBA, BMPA, BGJO, and IBGJO. It should be noted that all algorithms use the exact binary mechanisms. At the same time, BGJO is a binary version of the conventional GJO algorithm. IBGJO parameters are based on those of the conventional GJO algorithm, which has only one adaptive coefficient vector. Unlike other algorithms, conventional GJO and IBGJO require no additional tuning. The critical parameter choices for these algorithms are presented in Table 2, with specific values based on prior evidence of consistently strong performance in the literature for each algorithm, enabling effective feature comparison.

Moreover, because both the proposed IBGJO and these comparison algorithms are meta-heuristics, the size of the population and the number of iterations directly impact them. To guarantee that the comparison is fair, the population size and the number of algorithm iterations must be consistent. The population size and iteration count for each algorithm in this study are set to 20 and 200, respectively. Additionally, to prevent the experiment’s random bias, each algorithm is independently performed 30 times in these chosen datasets, as suggested by the central limit theorem. The experiment’s Intel(R) Core(R) I9-12900KF CPU and 64 GB of RAM were employed. Using Python 3.9.12 and the *K*NN [48] (*k* = 5) based on Euclidean distance measurement, we put the trials into practice. It is worth noting that a common approach has been employed in several previous works where 80% of the instances are used for training purposes, while the remaining instances are reserved for testing. Moreover, in the fitness function α and β are set to 0.99 and 0.01, respectively.

### 5.2. Feature Selection Results

This section presents the feature selection results of various algorithms in terms of average fitness function value, convergence speed, average accuracy, and average CPU time. Also, the best results are shown in bold.

#### 5.2.1. Performance Evaluation

To explicitly demonstrate the performance of various algorithms, the fitness function values achieved by those algorithms are shown in Table 3. Table 3 shows the numerical statistical results of each dataset’s average fitness function value and standard deviation (std) of different algorithms. For the average fitness values on 28 datasets, BCS, BGWO, BHBA, BMPA, BGJO, and IBGJO, they achieved the best performance on 3, 3, 5, 7, 9, and 14 datasets, respectively. This demonstrates our conjecture that BGJO may have a good exploration ability but lacks exploitation performance. Thus, by introducing the improved factors to conventional BGJO, the proposed improvement factors are practical. Compared with conventional BGJO, IBGJO has an advantage on average fitness value in 21 datasets. Moreover, IBGJO obtains the best stds of fitness values in 11 datasets, which means that IBGJO is more stable than others regarding feature selection.

**Figure 5:**
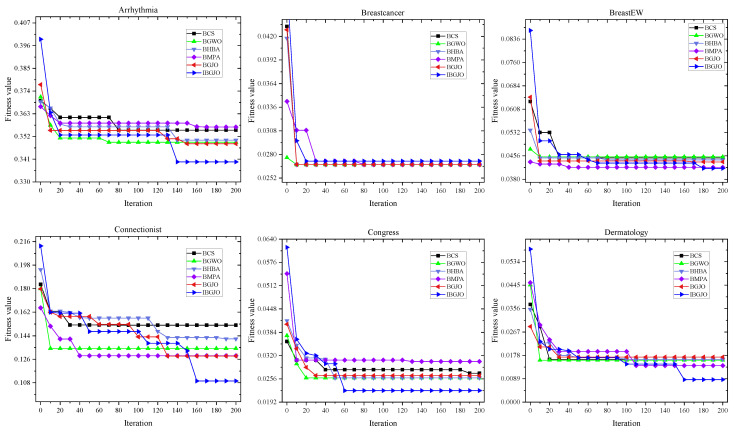

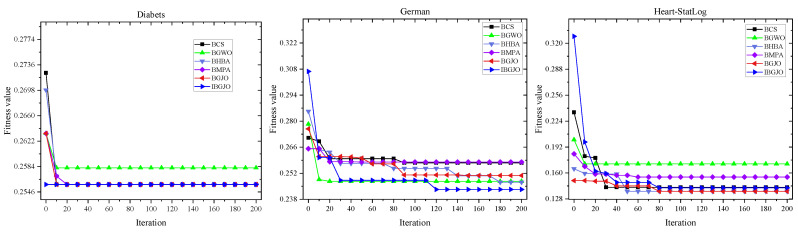
Convergence rates obtained by different algorithms (Part 1).

**Figure 6:**
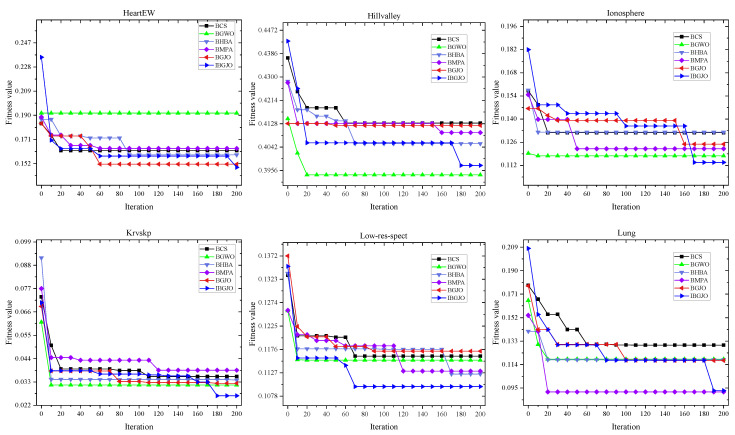

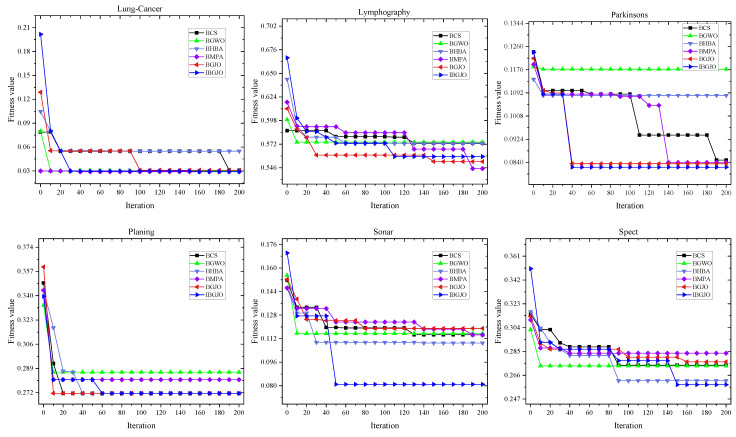
Convergence rates obtained by different algorithms (Part 2).

**Figure 7:**
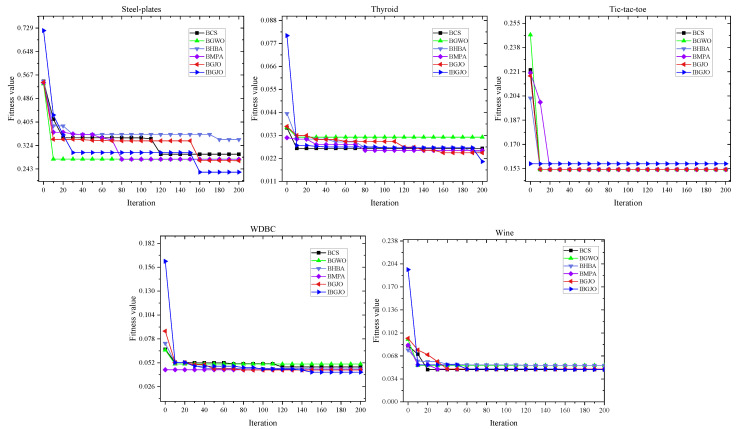

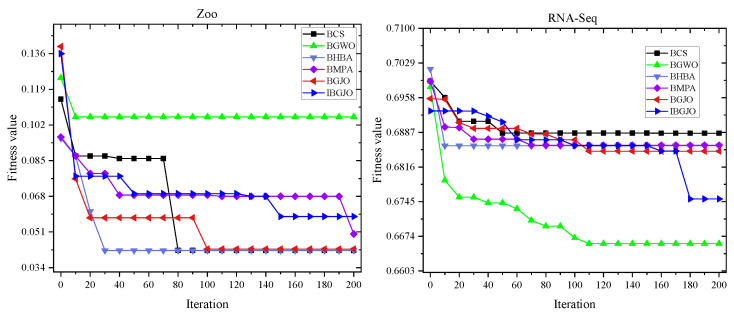
Convergence rates obtained by different algorithms (Part 3).

Due to space restrictions, many such figures are divided into three parts, and each curve is taken from the 15th test. The convergence rates of various algorithms used in the optimization processes are shown in Figure 5, Figure 6 and Figure 7. These figures demonstrate that the proposed IBGJO exposes the best curves on 20 datasets and has the best convergence capability compared to all other comparison algorithms. Overall, the proposed IBGJO performs better than other comparison algorithms for solving the formulated feature selection problem. Note that the effectiveness of different improved factors is further verified and discussed in Section 5.3.

#### 5.2.2. Features Selection Accuracy of Algorithms

The feature selection accuracy obtained by various algorithms is shown in Table 4. The IBGJO algorithm achieves the best average accuracies of feature selection results on 14 datasets. Moreover, IBGJO obtains better accuracy than conventional BGJO in 21 datasets. Thus, compared with other algorithms, the IBGJO algorithm has the best performance in terms of feature selection accuracies on these selected datasets. The reason could be that the improved factors can balance the exploration and exploitation abilities, improving the algorithm’s performance. However, it is crucial to recognize that achieving optimal results for accuracy and the number of selected features is a challenging tradeoff that varies across datasets.

Therefore, it can be concluded that the proposed IBGJO algorithm displays superior overall performance in feature selection across the selected datasets as compared to the other algorithms according to Table 4 and Table 5.

#### 5.2.3. Number of Selected Features

The counts of the selected features from the datasets acquired by various techniques are displayed in Table 5. Similar to the accuracy results, these tables likewise display the outcomes of numerical statistics. BMPA obtains the best average number of selected features in the majority of the datasets (20 of 28), which may be regarded as the best results in the tests compared to other algorithms. This is shown in Table 5. Meanwhile, the number of features of IBGJO has an advantage in 15 datasets compared to that of BGJO. It is important to note that there exists a tradeoff between accuracy and the number of selected features, making it challenging to achieve optimal results for both objectives in each dataset.

#### 5.2.4. Algorithm Execution Time

The average running time of all algorithms is shown in Table 6. Based on the data presented in Table 6, it is evident that the IBGJO has an advantage in algorithm execution time. IBGJO is experimented on 28 datasets and compares the performance of different feature selection algorithms. Among these 28 datasets, our algorithm converged in the least average time on 19 datasets. This means that our algorithm has higher efficiency and faster convergence and can select the best subset of features in less time, thereby improving the performance of the model. This result shows that our algorithm has higher practicability and feasibility in practical applications.

### 5.3. Effectiveness of the Improved Factors

**Figure 8:**
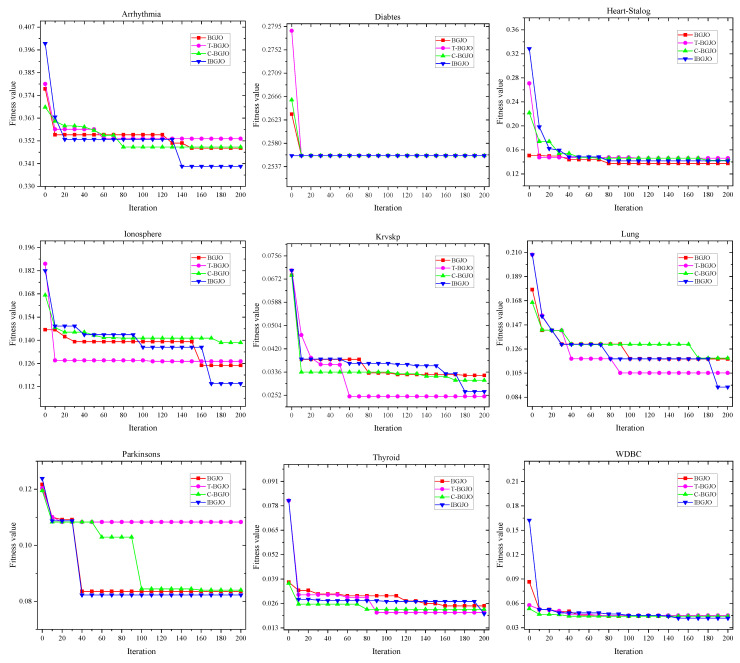
Convergence rate comparisons between conventional BGJO, T-BGJO, C-BGJO and IBGJO on different datasets.

In this section, we conduct experiments to evaluate the effectiveness of the introduced factors in IBGJO. To observe whether these factors can impove the performance of BGJO, we use BGJO, BGJO with CTM mechanism (T-BGJO), BGJO with CS (C-BGJO), and BGJO both with CTM mechanism and CS together (IBGJO) to solve the formulated feature selection problem, respectively. The tests are also conducted on the nine selected datasets: Arrhythmia, Diabets, Heart-StatLog, Ionosphere, Krvskp, Lung, Parkinsons, Thyroid and WDBC. The numerical findings generated by these abovementioned algorithms are listed in Table 7. Overall, all algorithms obtain the same results on the Diabets dataset. This may be because this dataset has the lowest solution dimension, making it easy to solve. Furthermore, the convergence rates of different improvement factors used in optimization are shown in Figure 8. The remaining outcomes are discussed in detail as follows.

#### 5.3.1. Effectiveness of the Chaotic Tent Map (CTM) Mechanism

It can be seen from Table 7 that compared with the traditional BGJO algorithm, the T-BGJO algorithm does not have many advantages in fitness function value or classification accuracy. However, T-BGJO could efficiently select a fewer number of features than BGJO. Therefore, CTM has the advantage in feature number over other improved factors with cosine similarity that can help IBGJO obtain better performance.

#### 5.3.2. Effectiveness of Cosine Similarity Position Update

In most datasets, especially medium-dimensional datasets, C-BGJO outperforms BGJO and T-BGJO in terms of accuracy of the fitness function values obtained, as shown in Table 7. This is due to the ability of the proposed CS position updating system to adaptively modify the searching scope to enhance BGJO’s exploration capabilities. Note that, compared with the location update mechanism of the conventional BGJO, the CS requires additional calculation in each iteration. However, the searchability of IBGJO will be more robust with CS. Therefore, it could increase the convergence time.

#### 5.3.3. Effectiveness of CTM and CS

It can be seen from Table 7 that the CTM mechanism effectively improves the representativeness and diversity of the initial population through a chaotic tent map and prevents the algorithm from falling into local optimum. Using CS as the jackal position update strategy in the golden jackal optimization algorithm can speed up the convergence speed of the algorithm and help IBGJO to converge faster. The combination of the two enhancement factors can effectively improve the algorithmic performance of BGJO in the field of feature selection.

To summarize, incorporating the two enhancement factors and binary mechanisms has effectively elevated the performance of the conventional BGJO algorithm and made it well-suited for feature selection. Furthermore, these components exhibit a complementary relationship. For instance, utilizing the CTM mechanism on small-sized datasets may cause the algorithm to encounter local optima frequently. Therefore, incorporating the CS is essential to address this problem.

### 5.4. Limitation of IBGJO

Although the experimental simulation results show that the proposed IBGJO algorithm outperforms some comparative algorithms, it still has some limitations. One limitation of the IBGJO algorithm is its sensitivity to parameter settings, requiring careful tuning for optimal performance. Additionally, the scalability of IBGJO to large-scale or high-dimensional datasets is a concern, as its computational complexity may become prohibitive. The generalization of IBGJO to different domains and problem types needs further exploration, as specific data characteristics may influence its performance. Furthermore, the interpretability of the selected feature subsets may not be guaranteed, as the algorithm prioritizes classification performance over intuitive feature combinations. The effectiveness and applicability of IBGJO can be improved by reducing the dimensionality of the feature set on the original dataset and then using the IBGJO algorithm for feature selection and optimizing the algorithm parameters.

## 6. Conclusions

The focus of this research work is to improve the classification performance of machine learning by addressing the issue of feature selection. An improved version of the BGJO algorithm, referred to as the IBGJO algorithm, is proposed to solve the feature selection problem. The IBGJO algorithm incorporates the CTM mechanism, CS location updating mechanism, and *S*-shape binary mechanism, designed to improve the performance of conventional BGJO and make it suitable for feature selection problems. Utilizing these improved factors allows the algorithm to balance its exploitation and exploration abilities while maintaining population diversity.

By using the improved factors, we can balance the development of the algorithm and its ability to explore different options while maintaining diversity within the population. We conducted experiments to test our proposed algorithm, IBGJO, on 28 well-known datasets and found that it outperformed other state-of-the-art algorithms such as BCS, BGWO, BHBA, BMPA and BGJO in terms of feature selection. We also evaluated the effectiveness of the improvement factors. We found that they helped to enhance the performance of the conventional golden jackal optimization algorithm. In the future, we plan to propose additional ways to update the location of the population and combine them with other evolutionary algorithms to tackle a broader range of optimization problems.

## Figures and Tables

**Table 1:** Benchmark datasets.

No.	Dataset	Instances	Features	Classses	Attribute Type	Area
D1	Arrhythmia	452	278	16	Categorical, Integer, Real	Life
D2	Breastcancer	699	10	5	Integer	Life
D3	BreastEW	569	30	2	Real	Life
D4	Congress	435	16	2	Categorical	Social
D5	Connectionist	208	60	2	Real	Physical
D6	Dermatology	366	34	6	Categorical, Integer	Life
D7	Diabets	768	8	7	Categorical, Integer	Computer
D8	German	1000	24	2	Categorical, Integer	Business
D9	HeartEW	270	13	2	Categorical, Real	Life
D10	Heart-StatLog	270	13	2	Categorical, Real	Life
D11	Hillvalley	606	100	2	Real	N/A
D12	Ionosphere	351	34	2	Integer, Real	Life
D13	Krvskp	3196	36	2	Categorical	Game
D14	Low-res-spect	531	102	9	Integer, Real	Physical
D15	Lung	72	326	7	Integer	Life
D16	Lung-Cancer	32	56	3	Integer	Life
D17	Lymphography	148	18	8	Categorical	Physical
D18	Parkinsons	1040	26	2	Integer, Real	Life
D19	Planning	182	13	2	Real	Computer
D20	Sonar	208	60	2	Real	Physical
D21	Spect	267	22	2	Categorical	Life
D22	Steel-plates	1941	27	7	Integer, Real	Physical
D23	Thyroid	7200	21	3	Categorical, Real	Life
D24	Tic-tac-toe	958	9	2	Categorical	Game
D25	WDBC	569	31	2	Real	Life
D26	Wine	178	13	3	Integer, Real	Physical
D27	Zoo	101	16	7	Categorical, Integer	Life
D28	RNA-Seq	802	16384	4	Real	Life

**Table 2:** Key parameters of different algorithms.

No.	Algorithm	Parameters
1	BCS	Discovery probability = 0.25, α = 1
2	BGWO	α = [2, 0]
3	HBA	β = 6, *C* = 2, $vec_{flag}$= [1, −1]
4	MPA	*P* = 0.5, FADS = 0.2
5	BGJO	$e_{0}$ = [−1, 1]
6	IBGJO	$e_{0}$ = [−1, 1]

**Table 3:** Average fitness function values obtained by different algorithms.

Datastet	BCS	BGWO	BHBA	BMPA	BGJO	IBGJO
	Fitness	Fitness	Fitness	Fitness	Fitness	Fitness
**D1**	0.3526 ± 0.0042	**0.3440** ± 0.0080	0.3489 ± 0.0052	0.3491 ± 0.0050	0.3477 ± 0.0041	0.3482 ± **0.0038**
**D2**	**0.0268** ± **0.0000**	0.0297 ± 0.0029	**0.0268** ± **0.0000**	**0.0268** ± **0.0000**	**0.0268** ± **0.0000**	0.0272 ± **0.0000**
**D3**	0.0452 ± 0.0015	0.0505 ± 0.0054	0.0440 ± 0.0006	**0.0433** ± 0.0009	0.0437 ± **0.0006**	**0.0433** ± 0.0009
**D4**	0.0274 ± 0.0025	0.0309 ± 0.0045	0.0256 ± 0.0020	0.0259 ± 0.0028	**0.0253** ± 0.0020	0.0269 ± **0.0018**
**D5**	0.1427 ± **0.0055**	0.1391 ± 0.0131	0.1382 ± 0.0072	0.1383 ± 0.0095	0.1378 ± 0.0070	**0.1362** ± 0.0095
**D6**	0.0165 ± 0.0017	0.0175 ± 0.0032	0.0149 ± **0.0013**	0.0169 ± 0.0017	0.0144 ± 0.0017	**0.0139** ± 0.0017
**D7**	**0.2557** ± **0.0000**	0.2588 ± 0.0042	**0.2557** ± **0.0000**	**0.2557** ± **0.0000**	**0.2557** ± **0.0000**	**0.2557** ± **0.0000**
**D8**	0.2523 ± 0.0037	0.2572 ± 0.0076	0.2517 ± **0.0027**	0.2535 ± 0.0032	**0.2503** ± 0.0030	0.2505 ± 0.0035
**D9**	0.1629 ± 0.0066	0.1733 ± 0.0104	0.1590 ± 0.0052	0.1587 ± 0.0044	0.1543 ± **0.0040**	**0.1537** ± 0.0046
**D10**	0.1463 ± 0.0054	0.1595 ± 0.0238	**0.1392** ± 0.0030	0.1414 ± 0.0049	0.1450 ± 0.0025	0.1440 ± **0.0024**
**D11**	0.4113 ± **0.0031**	**0.3995** ± 0.0064	0.4089 ± 0.0039	0.4076 ± 0.0041	0.4088 ± 0.0042	0.4079 ± 0.0042
**D12**	0.1374 ± **0.0048**	0.1326 ± 0.0112	0.1325 ± 0.0074	**0.1267** ± 0.0056	0.1307 ± 0.0058	0.1293 ± 0.0070
**D13**	0.0361 ± 0.0039	0.0373 ± 0.0053	0.0336 ± **0.0024**	0.0379 ± 0.0029	0.0325 ± 0.0025	**0.0316** ± 0.0026
**D14**	0.1170 ± **0.0016**	0.1148 ± 0.0035	0.1156 ± 0.0021	0.1154 ± 0.0019	0.1157 ± 0.0023	**0.1148** ± 0.0018
**D15**	0.1316 ± **0.0069**	0.1265 ± 0.0171	0.1230 ± 0.0093	0.1224 ± 0.0088	0.1216 ± 0.0098	**0.1197** ± 0.0107
**D16**	0.0386 ± 0.0113	0.0379 ± 0.0116	0.0327 ± 0.0072	0.0312 ± 0.0061	0.0302 ± 0.0006	**0.0301** ± **0.0005**
**D17**	0.5696 ± 0.0088	0.5837 ± 0.0167	**0.5618** ± 0.0069	0.5639 ± 0.0104	0.5651 ± 0.0055	0.5645 ± **0.0053**
**D18**	0.0988 ± 0.0116	0.1129 ± **0.0071**	0.0962 ± 0.0108	**0.0879** ± 0.0077	0.0903 ± 0.0104	0.0882 ± 0.0085
**D19**	0.2752 ± 0.0054	0.2992 ± 0.0233	0.2719 ± 0.0011	0.2749 ± 0.0052	**0.2716** ± **0.0000**	**0.2716** ± **0.0000**
**D20**	0.1147 ± **0.0057**	0.1094 ± 0.0137	0.1095 ± 0.0059	0.1092 ± 0.0080	0.1070 ± 0.0081	**0.1035** ± 0.0078
**D21**	0.2766 ± 0.0067	0.2792 ± 0.0121	0.2707 ± 0.0076	0.2736 ± 0.0075	**0.2678** ± 0.0071	0.2708 ± **0.0055**
**D22**	0.3193 ± 0.0324	0.4235 ± 0.1062	0.3286 ± 0.0344	0.2953 ± 0.0272	**0.2687** ± **0.0126**	0.2731 ± 0.0140
**D23**	0.0276 ± 0.0019	0.0314 ± 0.0049	0.0248 ± 0.0014	0.0252 ± 0.0016	0.0239 ± 0.0014	**0.0238** ± 0.0014
**D24**	**0.1523** ± **0.0000**	0.1541 ± 0.0098	**0.1523** ± **0.0000**	**0.1523** ± **0.0000**	0.1564 ± **0.0000**	0.1564 ± **0.0000**
**D25**	0.0462 ± 0.0018	0.0537 ± 0.0082	0.0443 ± 0.0007	**0.0437** ± 0.0007	0.0440 ± 0.0006	**0.0437** ± 0.0008
**D26**	0.0517 ± 0.0033	0.0621 ± 0.0128	0.0491 ± 0.0021	0.0494 ± 0.0021	**0.0483** ± 0.0005	**0.0483** ± **0.0004**
**D27**	0.0534 ± 0.0090	0.0794 ± 0.0180	0.0463 ± 0.0061	0.0512 ± 0.0070	**0.0442** ± **0.0047**	0.0634 ± 0.0051
**D28**	0.6865 ± **0.0024**	**0.6634** ± 0.0045	0.6844 ± **0.0024**	0.6826 ± 0.0029	0.6828 ± 0.0030	0.6817 ± 0.0.0027

**Table 4:** Classification accuracies and standard deviation achieved by different algorithms.

Datastet	BCS	BGWO	BHBA	BMPA	BGJO	IBGJO
	Accuracy	Accuracy	Accuracy	Accuracy	Accuracy	Accuracy
**D1**	0.6501 ± 0.0043	**0.6598** ± 0.0081	0.6539 ± 0.0053	0.6529 ± 0.0050	0.6551 ± 0.0042	0.6546 ± **0.0038**
**D2**	**0.9800** ± **0.0000**	0.9771 ± 0.0028	**0.9800** ± **0.0000**	**0.9800** ± **0.0000**	**0.9800** ± **0.0000**	0.9786 ± **0.0000**
**D3**	0.9594 ± 0.0010	0.9540 ± 0.0053	0.9598 ± **0.0004**	0.9599 ± 0.0007	0.9598 ± 0.0005	**0.9603** ± 0.0009
**D4**	0.9777 ± 0.0023	0.9748 ± 0.0041	0.9795 ± 0.0015	0.9790 ± 0.0025	**0.9797** ± 0.0016	0.9777 ± **0.0015**
**D5**	0.8617 ± **0.0054**	0.8662 ± 0.0130	0.8663 ± 0.0073	0.8654 ± 0.0095	0.8668 ± 0.0069	**0.8683** ± 0.0098
**D6**	0.9897 ± 0.0020	0.9900 ± 0.0031	0.9915 ± **0.0015**	0.9892 ± 0.0018	0.9921 ± 0.0017	**0.9923** ± .0018
**D7**	**0.7468** ± **0.0000**	0.7445 ± 0.0043	**0.7468** ± **0.0000**	**0.7468** ± **0.0000**	**0.7468** ± **0.0000**	**0.7468** ± **0.0000**
**D8**	0.7518 ± 0.0039	0.7476 ± 0.0077	0.7522 ± **0.0028**	0.7497 ± 0.0034	**0.7538** ± 0.0032	0.7535 ± 0.0037
**D9**	0.8394 ± 0.0059	0.8299 ± 0.0098	0.8430 ± 0.0048	0.8427 ± 0.0043	0.8473 ± 0.0038	**0.8496** ± **0.0037**
**D10**	0.8567 ± 0.0057	0.8444 ± 0.0235	**0.8646** ± 0.0035	0.8617 ± 0.0055	0.8569 ± **0.0018**	0.8575 ± 0.0019
**D11**	0.5907 ± **0.0031**	**0.6037** ± 0.0062	0.5931 ± 0.0039	0.5937 ± 0.0041	0.5932 ± 0.0042	0.5942 ± 0.0042
**D12**	0.8661 ± **0.0047**	0.8715 ± 0.0106	0.8710 ± 0.0072	**0.8758** ± 0.0054	0.8727 ± 0.0055	0.8738 ± 0.0066
**D13**	0.9699 ± 0.0040	0.9698 ± 0.0052	0.9725 ± 0.0024	0.9678 ± 0.0029	0.9736 ± **0.0024**	**0.9746** ± 0.0026
**D14**	0.8881 ± **0.0015**	**0.8910** ± 0.0035	0.8894 ± 0.0021	0.8887 ± 0.0022	0.8893 ± 0.0022	0.8902 ± 0.0018
**D15**	0.8733 ± **0.0070**	0.8788 ± 0.0174	0.8821 ± 0.0095	0.8817 ± 0.0090	0.8833 ± 0.0100	**0.8854** ± 0.0109
**D16**	0.9667 ± 0.0118	0.9675 ± 0.0115	0.9725 ± 0.0075	0.9733 ± 0.0062	**0.9750** ± **0.0000**	**0.9750** ± **0.0000**
**D17**	0.4302 ± 0.0087	0.4164 ± 0.0165	**0.4380** ± 0.0069	0.4353 ± 0.0103	0.4344 ± 0.0056	0.4356 ± **0.0056**
**D18**	0.9050 ± 0.0118	0.8905 ± **0.0072**	0.9073 ± 0.0112	**0.9155** ± 0.0080	0.9132 ± 0.0109	0.9153 ± 0.0090
**D19**	0.7275 ± 0.0057	0.7039 ± 0.0233	0.7312 ± 0.0013	0.7279 ± 0.0056	**0.7316** ± **0.0000**	**0.7316** ± **0.0000**
**D20**	0.8903 ± **0.0056**	0.8963 ± 0.0136	0.8956 ± 0.0061	0.8949 ± 0.0081	0.8978 ± 0.0081	**0.9016** ± 0.0079
**D21**	0.7265 ± 0.0066	0.7243 ± 0.0118	0.7322 ± 0.0077	0.7291 ± 0.0077	**0.7353** ± 0.0071	0.7323 ± **0.0054**
**D22**	0.6826 ± 0.0325	0.5773 ± 0.1076	0.6732 ± 0.0347	0.7060 ± 0.0273	**0.7332** ± **0.0125**	0.7289 ± 0.0140
**D23**	0.9765 ± 0.0018	0.9733 ± 0.0047	0.9791 ± 0.0014	0.9787 ± 0.0016	**0.9800** ± **0.0012**	**0.9800** ± **0.0012**
**D24**	**0.8563** ± **0.0000**	0.8543 ± 0.0103	**0.8563** ± **0.0000**	**0.8563** ± **0.0000**	0.8521 ± **0.0000**	0.8521 ± **0.0000**
**D25**	0.9585 ± 0.0015	0.9508 ± 0.0082	0.9596 ± **0.0000**	0.9596 ± 0.0005	0.9598 ± 0.0004	**0.9599** ± 0.0006
**D26**	0.9528 ± 0.0031	0.9428 ± 0.0125	0.9548 ± 0.0019	0.9546 ± 0.0021	**0.9556** ± **0.0000**	**0.9556** ± **0.0000**
**D27**	0.9524 ± 0.0090	0.9270 ± 0.0183	0.9597 ± 0.0065	0.9539 ± 0.0074	**0.9618** ± 0.0050	0.9412 ± **0.0046**
**D28**	0.3130 ± **0.0024**	**0.3379** ± 0.0046	0.3152 ± 0.0025	0.3160 ± 0.0029	0.3168 ± 0.0030	0.0.3179 ± 0.0028

**Table 5:** Number of selected features obtained by different algorithms with standard deviation.

Datastet	BCS	BGWO	BHBA	BMPA	BGJO	IBGJO
	Features	Features	Features	Features	Features	Features
**D1**	174.07 ± 3.05	200.67 ± 10.34	174.30 ± 8.14	**151.73** ± 11.51	175.03 ± 8.78	175.13 ± **7.79**
**D2**	7.00 ± **0.00**	7.07 ± 0.73	7.00 ± **0.00**	7.00 ± **0.00**	7.00 ± **0.00**	**6.00** ± **0.00**
**D3**	14.93 ± 2.43	14.67 ± 2.61	12.57 ± 1.82	**11.03** ± 1.87	11.80 ± 1.69	11.87 ± **1.31**
**D4**	8.60 ± 1.62	9.40 ± 1.43	8.47 ± 1.65	8.13 ± 1.50	8.37 ± 1.50	**7.80** ± **1.06**
**D5**	35.20 ± 4.37	39.67 ± 4.11	35.43 ± 3.14	**30.37** ± **3.11**	35.80 ± 3.78	34.63 ± 3.81
**D6**	21.63 ± 2.63	25.73 ± 2.14	22.23 ± **1.84**	**21.07** ± 2.10	22.37 ± 2.04	21.63 ± 2.24
**D7**	**4.00** ± **0.00**	4.73 ± 0.73	**4.00** ± **0.00**	**4.00** ± **0.00**	**4.00** ± **0.00**	**4.00** ± **0.00**
**D8**	15.83 ± 2.19	17.53 ± 2.00	15.30 ± 1.99	**13.73** ± 1.88	15.87 ± 2.13	15.53 ± **1.55**
**D9**	5.10 ± 1.19	6.33 ± 1.30	4.60 ± 0.88	**3.80** ± 0.79	4.07 ± **0.58**	6.23 ± 1.61
**D10**	5.70 ± 0.90	7.17 ± 1.04	6.60 ± **0.80**	5.90 ± 1.33	4.30 ± 1.21	**3.90** ± 0.96
**D11**	60.43 ± 6.32	71.83 ± 4.31	60.30 ± 4.45	**52.93** ± 5.30	60.60 ± 4.59	60.83 ± **4.18**
**D12**	16.53 ± 3.19	18.23 ± 2.78	16.20 ± 2.91	**12.70** ± **2.25**	15.77 ± 2.45	14.73 ± 3.02
**D13**	22.63 ± 2.12	26.57 ± 2.49	22.87 ± 2.03	**21.57** ± 2.73	22.87 ± **1.80**	23.13 ± 2.50
**D14**	61.77 ± 4.47	68.37 ± **4.31**	61.03 ± 4.56	**52.53** ± 4.79	60.67 ± 4.95	61.03 ± 5.63
**D15**	200.27 ± 11.05	208.70 ± 14.09	203.43 ± **8.02**	**170.80** ± 11.77	197.43 ± 11.71	202.13 ± 8.95
**D16**	31.53 ± 3.74	32.03 ± 3.82	30.57 ± **2.56**	**27.13** ± 3.20	30.70 ± 3.14	30.03 ± 2.64
**D17**	9.97 ± 1.70	10.77 ± 1.61	9.70 ± **1.32**	**8.77** ± 1.82	9.37 ± 1.38	10.20 ± 1.42
**D18**	10.87 ± 1.77	10.37 ± 2.01	10.27 ± **1.59**	**9.87** ± 1.86	10.00 ± 1.89	10.17 ± 1.86
**D19**	**6.57** ± 0.50	7.20 ± 1.42	6.93 ± 0.25	6.60 ± 0.49	7.00 ± **0.00**	7.00 ± **0.00**
**D20**	36.80 ± **2.79**	40.57 ± 3.88	36.53 ± 3.94	**31.17** ± 2.88	34.83 ± 3.27	36.30 ± 3.59
**D21**	12.83 ± **1.27**	13.87 ± 1.61	12.40 ± 1.45	**12.00** ± 1.83	12.63 ± 1.50	12.77 ± 1.55
**D22**	13.67 ± 2.01	13.37 ± 1.99	13.80 ± 2.66	**11.33** ± 1.78	12.40 ± **1.71**	12.63 ± 1.97
**D23**	8.97 ± 1.28	10.50 ± 1.20	8.63 ± 1.38	8.60 ± **0.95**	8.67 ± 1.32	**8.27** ± 1.20
**D24**	9.00 ± **0.00**	**8.93** ± 0.36	9.00 ± **0.00**	9.00 ± **0.00**	9.00 ± **0.00**	9.00 ± **0.00**
**D25**	15.67 ± 2.37	15.57 ± 2.42	13.50 ± 2.06	**11.57** ± 1.71	12.80 ± 1.61	12.23 ± **1.50**
**D26**	6.47 ± 0.76	7.03 ± 1.02	5.67 ± 0.60	5.83 ± 0.90	5.63 ± 0.67	**5.53** ± **0.57**
**D27**	10.00 ± 1.53	11.30 ± 1.49	10.17 ± 1.24	9.00 ± 1.21	10.27 ± **0.74**	**8.33** ± 1.58
**D28**	10,531.2 ± 442.10	12,951.73 ± 384.55	10,605.6 ± 75.16	8971.07 ± 365.87	10,548.87 ± 109.24	10,511.37 ± 164.68

**Table 6:** Average CPU time and standard deviation occupied by different algorithms (/s).

Datastet	BCS	BGWO	BHBA	BMPA	BGJO	IBGJO
	Time	Time	Time	Time	Time	Time
**D1**	94.33 ± **0.89**	588.37 ± 5.68	**92.33** ± 1.22	94.50 ± 1.10	449.13 ± 7.94	115.36 ± 12.48
**D2**	97.03 ± 1.37	121.97 ± 1.25	97.24 ± 0.08	96.84 ± **0.08**	111.89 ± 2.34	**79.24** ± 5.11
**D3**	**97.64** ± 2.10	157.52 ± 2.76	98.31 ± **0.73**	99.59 ± 1.05	139.61 ± 1.83	124.48 ± 10.02
**D4**	84.62 ± 0.30	116.75 ± 1.30	85.76 ± 0.54	83.37 ± **0.09**	108.24 ± 0.22	**72.70** ± 8.21
**D5**	389.48 ± 70.34	234.38 ± 46.57	423.26 ± 68.75	315.16 ± 75.90	471.42 ± 70.29	**85.94** ± **8.25**
**D6**	**69.31** ± 0.20	135.53 ± 1.93	71.70 ± 0.84	70.96 ± **0.09**	115.97 ± 1.54	89.77 ± 3.83
**D7**	100.13 ± 0.11	122.23 ± 1.44	102.85 ± 0.77	100.28 ± 0.11	112.59 ± **0.10**	**97.86** ± 14.02
**D8**	137.66 ± 0.98	192.54 ± 2.72	140.54 ± 1.04	135.96 ± **0.53**	174.12 ± 0.63	**125.01** ± 16.93
**D9**	65.53 ± 1.32	90.74 ± 0.51	65.83 ± 0.22	65.31 ± **0.04**	82.29 ± 0.38	**65.51** ± 8.20
**D10**	66.23 ± **0.03**	92.81 ± 0.78	67.01 ± 0.05	66.53 ± 0.08	61.54 ± 6.94	**58.60** ± 3.28
**D11**	96.49 ± 0.83	278.54 ± 3.70	97.79 ± 0.89	98.97 ± **0.49**	228.26 ± 4.15	**94.67** ± 19.25
**D12**	**75.61** ± 1.18	142.31 ± 2.82	75.95 ± 0.54	77.32 ± **0.26**	122.87 ± 1.38	104.94 ± 6.15
**D13**	664.07 ± 6.49	635.02 ± 6.44	578.17 ± **4.53**	707.68 ± 25.26	630.96 ± 15.30	**507.11** ± 89.94
**D14**	417.71 ± 32.64	192.66 ± **6.09**	242.64 ± 49.37	371.89 ± 77.69	123.42 ± 7.40	**120.51** ± 7.98
**D15**	52.69 ± **0.44**	637.31 ± 8.29	**51.72** ± 0.54	55.87 ± 0.67	473.91 ± 8.00	64.38 ± 6.19
**D16**	41.03 ± 0.42	144.48 ± 0.45	41.60 ± **0.05**	40.72 ± 0.06	111.79 ± 0.68	**28.65** ± 0.09
**D17**	53.97 ± 0.84	88.93 ± 0.49	54.70 ± 0.36	**53.14** ± **0.06**	54.37 ± 11.06	58.93 ± 10.84
**D18**	122.17 ± 21.75	126.41 ± 39.18	117.47 ± 9.85	122.53 ± 29.08	115.12 ± **7.12**	**75.68** ± 7.61
**D19**	86.25 ± 20.06	120.99 ± **0.71**	107.40 ± 20.81	105.63 ± 21.03	100.11 ± 23.92	**56.36** ± 8.58
**D20**	60.99 ± 0.57	173.97 ± 2.53	**60.23** ± **0.06**	61.72 ± 0.83	138.64 ± 0.14	71.42 ± 12.01
**D21**	65.07 ± **0.09**	107.92 ± 1.98	65.81 ± 0.10	66.57 ± 0.38	97.23 ± 1.23	**50.55** ± 0.75
**D22**	232.57 ± 22.23	478.93 ± 141.93	249.02 ± 76.10	304.31 ± 39.79	281.79 ± 38.30	**78.64** ± **2.29**
**D23**	1181.20 ± **68.71**	1204.65 ± 108.30	1204.61 ± 86.49	1178.79 ± 69.78	1186.05 ± 72.15	**576.16** ± 77.74
**D24**	123.54 ± 1.21	155.56 ± 2.17	128.09 ± 0.16	121.07 ± **0.15**	119.57 ± 10.52	**88.02** ± 0.27
**D25**	**96.00** ± 0.86	159.74 ± 2.28	97.21 ± 0.80	98.47 ± **0.63**	138.97 ± 1.37	126.37 ± 5.85
**D26**	55.53 ± 0.10	81.56 ± 0.62	57.16 ± **0.04**	55.83 ± 0.07	50.07 ± 4.24	**42.41** ± 0.05
**D27**	51.53 ± 1.03	79.69 ± 0.18	50.41 ± 0.05	49.34 ± **0.04**	70.65 ± 0.14	**45.02** ± 2.34
**D28**	9275.34 ± 1507.77	10,200.77 ± 2736.28	9238.49 ± 1519.09	8351.13 ± 1349.94	3107.74 ± **482.36**	6432.33 ± 801.44

**Table 7:** Average results obtained by different improved factors of IBGJO.

		D1	D7	D10	D12	D13	D15	D18	D23	D25
**BGJO**	Accuracy	0.6551	**0.7468**	0.8569	0.8727	0.9736	0.8833	0.9132	**0.9800**	0.9598
	Feature.N	175.03	**4.00**	4.30	15.77	22.87	197.43	10.00	8.67	12.80
	Fitness	0.3477	**0.2557**	0.1450	0.1307	0.0325	0.1216	0.0903	0.0239	0.0440
	Time	449.13	112.59	61.54	122.87	630.96	473.91	115.12	1186.05	138.97
**T-BGJO**	Accuracy	0.6543	**0.7468**	0.8519	0.8728	0.9733	0.8829	0.9102	0.9794	0.9598
	Feature.N	**173.53**	**4.00**	5.27	**14.60**	23.07	198.47	**9.60**	8.47	12.80
	Fitness	0.3485	**0.2557**	0.1507	0.1302	0.0328	0.1220	0.0931	0.0244	0.0440
	Time	152.07	**93.60**	**56.72**	**80.57**	607.59	**41.60**	**61.62**	**539.04**	**104.90**
**C-BGJO**	Accuracy	**0.6553**	**0.7468**	0.8574	0.8727	0.9742	0.8838	0.9122	0.9797	0.9597
	Feature.N	174.90	**4.00**	4.37	15.53	**22.60**	**197.07**	10.33	8.57	12.90
	Fitness	**0.3476**	**0.2557**	0.1445	0.1306	0.0318	0.1212	0.0914	0.0241	0.0441
	Time	119.85	100.28	63.25	107.20	**503.21**	59.36	73.73	550.47	127.88
**IBGJO**	Accuracy	0.6546	**0.7468**	**0.8575**	**0.8738**	**0.9746**	**0.8854**	**0.9153**	**0.9800**	**0.9599**
	Feature.N	175.13	**4.00**	**3.90**	14.73	23.13	202.13	10.17	**8.27**	**12.23**
	Fitness	0.3482	**0.2557**	**0.1440**	**0.1293**	**0.0316**	**0.1197**	**0.0882**	**0.0238**	**0.0437**
	Time	**115.36**	97.86	58.60	104.94	507.11	64.38	75.68	576.16	126.37

## Data Availability

Not applicable.

## Abbreviations

The following abbreviations are used in this manuscript:

Acc	Average accuracy
CS	Cosine similarity
EA	Evolutionary algorithm
FS	Feature selection
LD	Linear dichroism
MJ	Male golden jackal
FMJ	Female golden jackal
Std	Standard deviation
